# A 15-year prospective study of shift work and disability pension

**DOI:** 10.1136/oem.2007.036525

**Published:** 2008-01-15

**Authors:** F Tüchsen, K B Christensen, T Lund, H Feveile

**Affiliations:** National Research Centre for the Working Environment, Copenhagen, Denmark

## Abstract

**Objective:**

To estimate the hazard ratio for disability pension associated with shift work.

**Methods:**

Cohorts of shift and day workers were identified in three waves of the Danish Work Environment Cohort Study and followed up for incidence of disability pension in a national register of social transfer payment. A total of 3980 female and 4025 male employees were included in the cohorts. Information about shift work status, age, smoking habits, body mass index and ergonomic work environment were updated according to responses in subsequent waves of the survey when possible. Respondents reporting shift work were classified as shift workers in the following waves as well. Respondents were followed in the register from the time of first interview and were censored at the time of their 60th birthday, emigration, death or end of follow-up (18 June 2006). The authors used the Cox proportional hazards model to estimate hazard ratios for incidence of disability pension and 95% confidence intervals.

**Results:**

The authors observed 253 new disability pensions among women and 173 among men during 56 903 and 57 886 person-years at risk respectively, Among women, shift work predicted disability after adjustment for age, general health and socioeconomic status HR 1.39 (95% CI 1.07 to 1.82). After further adjustment for body mass index, smoking habits, socioeconomic status and ergonomic exposures the association remained statistically significant HR 1.34 (95% CI 1.02 to 1.75). Shift work was not associated with disability among men.

**Conclusion:**

Shift work might be moderately associated with disability pension among women; however, more powerful studies are needed to establish the possible association.

Shift work has been identified as a potential risk factor for heart disease,^1^ breast cancer,^2^ gastric ulcer^3^ and injuries.^4^ Bridge construction workers who worked 84-h weeks and other rotas with overtime had a relative risk of becoming disabled rising from less than one in the youngest age group, to approximately three among those over 45 years of age.^5^ It is, therefore, quite plausible that shift work is a risk factor for disability pension, but to what extent is not known. Furthermore, a recent study showed that employees in industries known to work shifts and long hours, such as transport, hotels and restaurants, were more likely to retire with a disability pension.^6^

Disability pension (early age pension) is granted by local community boards, taking both individual workability and community employment opportunities into consideration. Therefore disability rates should not be seen purely as indicators of health—rather they express important changes in quality of life for the individual and a loss of skills and creativity for society. Working conditions have a strong influence with respect to the granting of disability pension because all possibilities for employment and rehabilitation should be tested before the claim is accepted. Albertsen *et al* found that both men and women had a higher risk of disability pension when they had to work standing or if they smoked. Women were also more likely to get disability pension if they were public employees, had low job security, or low social support at work. The possible effect of shift work was not included in their analysis.^7^ A Danish age-matched case-control study among unskilled male workers found no association between shift work and disability pension.^8^

The aim of the present study was to estimate a possible excess risk of disability among men and women on shifts.

## MATERIAL AND METHODS

This study is based on a merger of work environment exposure information from the Danish Work Environment Cohort Study (DWECS) and information about granted disability pension from a national register of social transfer payment: the DREAM register.^9^ DWECS^10^ has a split panel design: in 1990 a simple random sample consisting of people aged 18–59 years on 1 October 1990 was drawn from the Danish centralised civil register. In 1995 and 2000 additional panels were drawn to adjust for immigration and for the ageing of the 1990 panel. The size of each panel reflects the proportion of the relevant groups of the total population.

In each wave, people who had been employees within two months prior to the interview were asked questions regarding work environment and health behaviour. The 1990 sample had 8664 participants (response rate 90%); 5701 were employees. The combined 1995 sample had 8583 participants (response rate 80%); 5369 were employees. The combined 2000 sample had 8583 participants (response rate 75%); 5366 were employees. In total 8475 employees—4288 men and 4187 women—were included in this study.

The study includes cohorts of shift workers who, in any of the three waves of the DWECS, stated that they worked one of the following work schedules: irregular working hours (including morning work), two-shift or fixed evening and three-shift or fixed night. Shift workers were compared with permanent day workers. Those reporting to be working shift were considered shift workers for the remainder of the study period regardless of later shift work status.

Subjects were followed in the DREAM register from the time of first interview and were censored at the time of their 60th birthday, emigration, death or end of follow-up (18 June 2006). DREAM contains information on all granted disability pensions to people employed both in the public and private sector. The municipal authorities grant disability pension, based on assessment of somatic and mental health and work ability: the individual’s work ability has to be permanently reduced to such a degree that return to work is unlikely. By law, it is possible for the recipient to work part-time while receiving disability pension, and also to return to work later. However, these options are rarely used, and in reality disability pension represents a permanent departure from the labour market in Denmark.^11^ DREAM does not include information about the underlying diagnosis for disability pension, and therefore the study does not allow the investigation of associations between shift work and the underlying diagnosis for granted disability pension. When individuals reach 60 there are various age pension schemes available instead of disability pension.

We used the Cox proportional hazards model to estimate hazard ratios for incidence of disability pension and 95% confidence intervals for each gender separately. Confounder information was entered in the model as time-varying variables: those participating again in a subsequent interview were followed with updated confounder information. Those not participating in subsequent interviews were followed with existing confounder information, except age which was updated in all. Adding a time-dependent interaction term between the exposure variable and the logarithm of follow-up time confirmed that the proportional hazards assumption was justified. In the first model we controlled for general health (GH) socioeconomic status (SES) and age; in the second model we also controlled for body mass index (BMI) and smoking habits; and in the third model we controlled for ergonomic exposures—that is, physically hard work, working with hands above the shoulders, working in a squatting or kneeling position and having repetitive work tasks.

Prevalent cases were controlled by means of a dichotomous question regarding GH. SES was controlled for by means of a variable based on employment grade, job title and education.

Body mass index was calculated from self-reported information on weight and height and categorised according to the standard classification of the National Institutes of Health (BMI <18.5, underweight; BMI 18.5–24.9, normal; BMI 25–29.9, overweight; BMI ⩾30, obese). Regarding smoking status the population was divided into heavy smokers (⩾15 cigarettes/day), moderate smokers (<15 cigarettes/day), ex-smokers and non-smokers.

The ergonomic work environment was assessed with questions on physically hard work (“Is your work so physically hard that you breathe faster?”), working with hands above the shoulders (“Do you work with your hand lifted to shoulder height or higher?”), and working in a squatting or kneeling position (“Does your work imply that you squat or kneel?”). Those responding “Seldom” or “Never” where categorised as unexposed, whereas those responding between “¼ of working hours” and “Almost all work hours” where categorised as exposed. Repetitive work tasks were assessed using a single question (“Does your work require that you repeat the same work tasks many times per hour?” Response options were categorised as follows: high, “Almost all working hours” or “¾ of working hours”; medium, “½ of working hours” or “¼ of working hours”; low, “Seldom” or “Never”.

## RESULTS

A total of 3980 women and 4025 men were without missing data and entered this study. During 56 903 person-years at risk for women and 57 886 years for men, we observed 253 new disability pensions among women and 173 among men. The incidence rate per 1000 person-years was for shift workers/day workers—women, 5.64/4.16; men, 3.25/3.02. For women and men we found hazard ratios for disability pension of 1.39 and 0.92 respective after adjustment for age, GH and SES. For both genders together the hazard ratio was 1.19 (table 1).

**Table 1:** Person-years at risk, hazard ratios and 95% CI for disability pension among Danish adults under 60 years of age

Gender (n)	Work period	Person-years at risk	Cases	Adjusted for age and GH and SES	Adjusted for age, GH, SES and BMI, smoking status	Adjusted for age, GH, SES, BMI, smoking status and ergonomic exposures
				HR (95% CI)	HR (95% CI)	HR (95% CI)
Women (3980)	Shift	13806	79	1.39 (1.07 to 1.82)	1.36 (1.04 to 1.78)	1.34 (1.02 to 175)
	Day	43097	174	1.00	1.00	1.00
Men (4025)	Shift	13000	41	0.92 (0.65 to 1.31)	0.94 (0.66 to 1.34)	0.93 (0.65 to 1.33)
	Day	44886	132	1.00	1.00	1.00
All	Shift	26806	120	1.19 (0.97 to 1.48)	1.19 (0.96 to 1.47)	1.18 (0.96 to 1.46)
	Day	87983	306	1.00	1.00	1.00

GH, general health; SES, socioeconomic status; BMI, body mass index.

The hazard ratio between shift work and disability pension when adjusting for age, GH, SES, BMI, smoking status and ergonomic exposures were 1.34 for women, 0.93 for men and 1.18 for both genders together (table 1). 

## DISCUSSION

When controlling for age, general health and SES we found an increased hazard for disability pension among women but not men. The increased hazard for women remained significant after controlling for general health, SES, BMI, smoking status and ergonomic exposures.

Our study has some limitations and certain strengths that should be taken into consideration. First, we have no information about how long individuals have worked shifts except for the three point measures in 1990, 1995 and 2000, and it is possible that people in the reference group worked shifts before or in between the measurements. That is likely to dilute the exposure contrast and our results are conservative for that reason. Second, we have collapsed all shift systems into one group in order to obtain a sufficient number. What we miss is, again, some of the contrast in exposure. Reforms of the Danish disability pension schemes were implemented during the study period in order to restrict access to permanent disability pension. This could imply that the strength of the association between shift work and disability pension could vary during the follow-up period: shift work would probably be more strongly associated with disability pension in the latter part of the study period, than with disability pension in the beginning of the study period.

Known risk factors for disability pension are prevalent disease, low self-perceived health, health risk behaviours, higher age, female sex, low SES and unemployment. In addition, stressful characteristics of work have been found to be associated with a risk of disability pension. Thus, the crude association observed between shift work and disability pension might well be due to baseline differences not controlled for. On the other hand one must bear in mind that SES is a complex strong indicator variable. Therefore adjustment for both SES and specific exposure variables may lead to over-adjustment. If we have adjusted partly for chains in the causal path we may also underestimate the true risk. Our adjustment for self-reported general health may also be an incomplete adjustment for prevalent disease because many with disabling chronic conditions still claim to have an excellent health. If so, our estimates are conservative.

Our study also has certain strengths. First, exposure information was collected before disability retirement and information bias is therefore ruled out. Our study is nationwide and representative for all first-time disability pensions among shift, ex-shift and day workers in Denmark. Another important strength is that no individuals were lost to follow-up.

**Main message**

Shift work may carry a moderately increased risk of disability among women.

**Policy implication**

More powerful studies are needed before final conclusions can be reached regarding associations between shift work, gender and disability.

Shift work is suspected to cause heart disease, breast cancer, peptic ulcer, sleep disturbances, compromised pregnancy outcome and accidents.^12^ It is therefore not surprising if the incidence of disability retirement is higher among shift workers, but we have no knowledge about why women should be more vulnerable to shift work than men as our results suggest.

In conclusion we found that shift work may increase the risk of later disability pension and might have a different effect on the risk of disability for men and women. However, more powerful studies are needed before a final conclusion can be reached.
